# Removal of the hazardous, volatile, and organic compound benzene from aqueous solution using phosphoric acid activated carbon from rice husk

**DOI:** 10.1186/s13065-014-0052-5

**Published:** 2014-09-03

**Authors:** Sobhy M Yakout

**Affiliations:** Biochemistry Department, College of Science, King Saud University, P.O. Box, 2455, Riyadh, 11451 Kingdom of Saudi Arabia; Hot Laboratories Centre, Atomic Energy Authority, Cairo, 13759 Egypt

**Keywords:** Benzene, Adsorption kinetics, Rice husk, Chemical activation

## Abstract

**Background:**

Benzene is one of the most hazardous organic pollutants in groundwater. The removal of benzene from water is very important from a health point of view and for environmental protection. In this study, benzene adsorption kinetics was investigated using phosphoric acid activated carbon, prepared from rice husk.

**Results:**

An initial rapid uptake of benzene was observed and became almost constant after 40 minutes of contact. Kinetic data was analyzed using pseudo first order, pseudo second order, and Elovich equations. Kinetic data was well fitted to pseudo-second order models (R^2^ = 0.98), indicating chemisorption. Results from intraparticle diffusion and Boyed models indicate that particle diffusion is the most probable operating mechanism and does not control the kinetics of benzene sorption. A comparative study on the benzene adsorption revealed that the rice husk carbon (RHC) had better benzene adsorption capacity (365 mg/g) as compared to other adsorbents.

**Conclusions:**

In conclusion, we have demonstrated that rice husk carbons are efficient benzene adsorbents and that they possess a good potential for benzene removal in wastewater treatment.

**Graphical Abstract:**
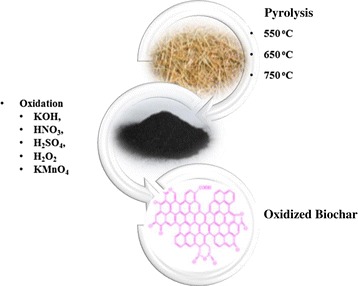
Phosphoric acid activated carbon from rice husk and benzene adsorption mechanism.

## Background

Aromatic compounds such as benzene are classified as flammable, toxic, carcinogenic, and/or mutagenic agents [1]. These compounds, however, are often employed in chemical processes as raw materials or even as solvents. The removal of these organic pollutants from the resulting wastewater is critical to ensure the safety of our water supplies.

A considerable effort has been dedicated in the past years concerning the removal of these compounds from wastewater. Several methods have been proposed and developed, and the most extensively used is the adsorption process [2]. Adsorption on activated carbon is a proven, reliable technology for the removal of small quantities of soluble organic compounds from water or wastewater. In addition, activated carbon adsorption has been cited by the US Environmental Protection Agency as one of the best available environmental control technologies [3].

Rice husk (RH) is an agricultural by-product abundantly available in rice producing countries. They are the natural sheaths that form on rice grains during their growth. The estimated annual world rice production is about 571 million tons, resulting in approximately 140 million tons of rice husk available annually for utilization.[4] In Egypt, rice husk is one of the most important agricultural residues in quantity [5]. Traditionally, rice husks have been disposed in landfills or used as a bedding material for animals. However, industrial applications of this material are limited. Most of the rice husks are left in rural areas or rice processing plants, with little application in industry. The most common method for the disposal of rice husks (RH) is incineration on farms. This produces ash, fumes, and toxic organic gases, leading to serious air pollution. The rice husks with hard surfaces, high silicon content, and small bulk density cannot be easily decomposed by bacteria. So, it is a dramatic source of pollution and leads to a series of environmental problems including eutrophication [6]. If we cannot utilize an appropriate approach to exploit rice husk, the hazardous materials will be released into the environment, thereby causing numerous problems that cannot be ignored [7]. The processing and transformation of rice husks into activated carbon with good adsorption properties would alleviate problems of disposal and management of these waste by-products, while producing value-added products from rice husks for water and wastewater treatment etc., to expand the carbon market. According to recent reviews on activated carbon from rice husks, chemical activation with KOH, NaOH, Na_2_CO_3_, K_2_CO_3_ H_3_PO_4_ at different temperatures for various times were used for activation [8]. Previous research has demonstrated the ability of rice husk-based activated carbon for many metal ions and organic molecules from aqueous phase adsorption.

Today, adsorption kinetic study still attracts considerable interest because of its particular significance in adsorbent evaluation and application, and more deliberate but complicated models will be proposed on the basis of the adsorption mechanism and diffusion analysis. In this work, analyses and batch adsorption experiments have been carried out to characterize and to understand the adsorption mechanism by modeling the adsorption kinetic. The aim of this paper is to study the possibility of the removal of benzene by rice husk activated carbon. A kinetic study according to different models has been applied. Using batch studies, the rate constants and the reaction order have been calculated.

## Results and discussion

The rice husk used was a locally available material in Egypt. It had the following approximate dimensions [9]: 8–10 mm long, 2.0–2.5 mm wide and 0.1–0.15 mm thick. The RH sample was analyzed according to standard ASTM:D-3172-73 method and was found to contain 64.3% volatile matter, 15.9% fixed carbon and 19.8% ash. Moreover, the main constituents of the metallic residues (ash content) are: silica 94.5%, calcium oxide 0.25%, magnesium oxide 0.23%, sodium oxide 0.78%, potassium oxide 1.10%, ferric oxide trace (<0.5), phosphorous pentoxide 0.53% and sulfur oxide 0.6%.[9]

### Adsorption profile

**Figure 1:**
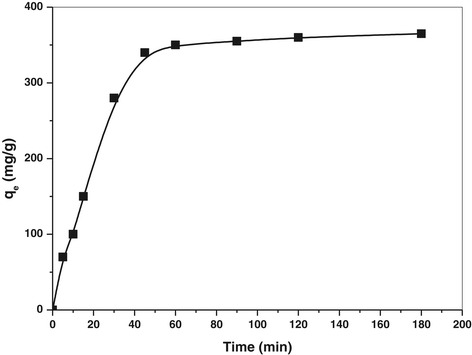
**Benzene adsorption profile on RHC.**

The adsorption profile of benzene uptake with time is shown in Figure 1, where a plot of amount of benzene adsorbed versus contact time is depicted. The removal curves are single, smooth, and continuous leading to saturation, suggesting possible monolayer coverage of benzene molecules on the surface of the RHC adsorbent. Benzene removal increases with time and attains equilibrium at 40 min. A short equilibrium time is one of the important considerations for economical wastewater treatment applications. A quick glance at Figure 1 reveals two sections of the uptake profile which are (i) the initial rapid uptake of benzene between 0 and 30 min and (ii) a more gradual process that comes to an equilibrium state after 40 min of contact. No further uptake of benzene by RHC was observed after the equilibrium time of 40 minutes. The initial rapid adsorption of benzene molecules on RHC is due to the availability of a larger number of vacant adsorption sites for the benzene of the bulk solution. The subsequent slower adsorption is likely because of the competition among the benzene for the limited number of vacant adsorption sites. Thus, the driving concentration gradient between the bulk solution and the solid surface is the main factor controlling the kinetics of the system. A similar trend of rapid initial and subsequent slower adsorption was reported by many researchers [3,10,11]. The initial slopes of Figure 1 show that the uptake rate of benzene by RHC is greatly dependent on the initial benzene concentration. The rapid adsorption rates indicate that the predominant mechanism for benzene adsorption on RHC is film diffusion, which is activated by the high concentration difference between the bulk solution and the pores of the adsorbent.

### Kinetic study

Every adsorption process may follow one or a combination of different patterns such as chemical reactions, diffusion control, and mass transfer. The analysis of experimental data at various times makes it possible to calculate the kinetic parameters and to take some information for designing and modeling the adsorption processes. To understand the adsorption mechanism of RHC for benzene, the adsorption kinetics were investigated using pseudo first-order [12], pseudo-second-order [13], and the Elovich kinetic equation [14] models. Listed models are respectively presented by the following equations:

1 $$ log\left({q}_e-{q}_t\right)= \log \left({q}_e\right)-\frac{k_1}{2.303}t $$

2 $$ \frac{t}{q_t}=\frac{1}{k_2{q}_e^2}+\frac{t}{q_e} $$

3 $$ {q}_t=\frac{1}{\beta }1\mathrm{n}\left(\alpha \beta \right)+\frac{1}{\beta } lnt $$

where q_e_ and q_t_ (mg g^−1^) and are the amount of benzene adsorbed at equilibrium and time t respectively, k_1_ (min^−1^) and k_2_ (g/mg min) are the rate constant of pseudo-first and pseudo-second-order rate adsorption, α (mg g^−1^ min) is the initial adsorption rate and *β* (g/mg) is the desorption constant. The experimental kinetic results modeled above mention situations and their estimated parameters are shown in Table 1. The conformity between experimental data and the model predicted values was expressed by the correlation coefficients. Another judgment is based on the agreement between experimental and theoretical experimental value. The result indicates that the pseudo-second-order model (R^2^ = 0.98) is more suitable than the pseudo first-order kinetic model (R^2^ = 0.92) and the Elovich model (R^2^ = 0.9) for benzene adsorption on RHC, and that the adsorption complies with the pseudo-second-order reaction. The calculated q_e_ values obtained from the first-order kinetic model do not give responsible values, which are too low compared with experimental qe values. Estimated q_e_ values of pseudo-second-order model accurately predict the adsorption kinetics over the entire working times. Therefore, this model has enough sufficiency for precise and acceptable predictions of the kinetics of benzene adsorption onto RHC. This suggested that the overall rate of the adsorption process is most likely to be controlled by the chemisorption process [15] and that the rate of reaction is directly proportional to the number of active sites on the surface of the adsorbent. According to the pseudo second order model, the adsorption rate dq_t_/dt is proportional to the second order of (q_e_-q_t_). Since RHC have relatively high equilibrium adsorption density q_e_, the adsorption rates become very fast and the equilibrium times are short. Such short equilibrium times coupled with high adsorption capacity indicate a high degree of affinity between adsorbate molecules and carbon surfaces [16].

**Table 1:** **Kinetic parameters of benzene adsorption onto RHC**

**Model**	**Parameter**	**Value**
**Experimental date**	**q**_**e,exp**_ **(mg/g)**	**365**
	**K**_**1**_ **(g/mg min)**	**38.2×10** ^**−3**^
**Frist-order**	**q**_**e**_ **(mg/g)**	**316**
	**R** ^**2**^	**0.92**
**Second-order**	**K**_**2**_ **(g/mg min)**	**0.125×10** ^**−3**^
	**q**_**e**_ **(mg/g)**	**400**
	**R** ^**2**^	**0.98**
	**α(mg/g min)**	**4.4**
**Elovich**	**β (g/mg)**	**10.4×10** ^**−3**^
	**R** ^**2**^	**0.9**
	**K**_**id**_ **(mg/g min** ^**1/2**^ **)**	**31.4**
**Intra-particle diffusion**	**C**	**114.7**
	**R** ^**2**^	**0.94**

### Adsorption kinetics mechanism

The adsorption in which an adsorbate is adsorbed from the liquid phase onto the adsorbent particles involves three steps. The first step is the film diffusion stage, in which the adsorbate diffuses from the aqueous bulk solution to the external surface of sorbent particles. The second step is called the intraparticle diffusion stage, during which the adsorbate diffuses from the outer surface into the particle interior through the pores. The third step is the adsorption of the adsorbate at an internal sit. The overall rate of adsorption depends on the slowest stage in the above process. The third step is very rapid in nature and cannot be taken into account for the rate determining step [17]. The pseudo-first-order, pseudo-second-order, and the Elovich kinetic models cannot generally identify the diffusion mechanism and rate-controlling steps that affect the adsorption. In order to understand the rate controlling step, the experimental data was subjected to the models that follow.

### Intraparticle diffusion study (Weber and Morris model)

**Figure 2:**
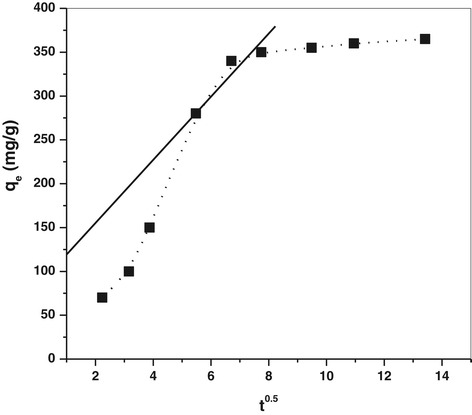
**Intra particle diffusion for the adsorption of benzene on RHC.**

The model of intraparticle diffusion is of great concern because it plays a significant role in the rate determining step in the equilibrium adsorption process. The rate constants of intraparticle diffusion (k_id_) are determined by using the following equation [18] and are depicted in Table 1

4 $$ {q}_t={k}_{id}{t}^{0.5} $$

where k_id_ (mg/g.min^0.5^) represents the intraparticle diffusion rate constant and its values are obtained from a plot of q vs. t^0.5^ (Figure 2). The ‘C’ values are obtained from the intercept of the plot and represent a constant depicting resistance to mass transfer in the boundary layer. The larger the value of C, the greater is the boundary layer effect. For pure intraparticle diffusion to take place, the plot of q vs. t^0.5^ should be linear, passing through the origin with no intercept. However, if the plot shows multilinearity, then the adsorption process may be controlled by the combination of film and intraparticle diffusion, i.e. more than one step is involved in the adsorption process [19].

When the benzene kinetic data was analyzed according to the intraparticle diffusion described by Weber and Morris [20], it was observed that the adsorption profile can be separated into three sections as shown in Figure 2. According to Onal et al. [21], the three stages involves an initial sharper portion, which is attributed to the diffusion of adsorbate through the solution to the external surface of the adsorbent or the boundary layer diffusion of solute molecules. The second portion describes the gradual adsorption stage, in which intraparticle diffusion may be rate limiting. The third stage involves the diffusion of sorbate particles to adsorption sites. The adsorption mechanism can frequently be controlled by multi-diffusion steps, involving film diffusion and intra-particle diffusion [22]. It may be concluded that surface adsorption and intraparticle diffusion were concurrently operating during the RHC and benzene interactions [23].

The intercepts obtained by the extrapolation of the second portion of the plots back towards the y-axis show that intraparticle diffusion is not the only controlling step of the process because the plot does not pass through the origin. This deviation may be due to the differences in the mass transfer rate in the initial and final stages of adsorption. The intraparticle diffusion rate constant (k_id_) obtained from the linear portions of the plots is depicted in Table 1. Similar observations were reported by many researchers while studying the adsorption behavior of different substrates.

### Boyd model

In order to characterize the actual rate-controlling step involved in the adsorption of benzene by RHC, the adsorption data was further analyzed according to the kinetic expression developed by Boyd et al. [24]. If the rate-determining step is diffusion through the adsorbent, then the following equation is valid:

5 $$ F=1-\left(6/{\pi}^2\right) \exp \left(-{B}_t\right) $$

and

6 $$ F=\frac{q_t}{q_{\infty }} $$

Where q_t_ represents the amount of benzene adsorbed (mg/g) at any time t (min), q_α_ is the amount of benzene adsorbed at infinite time (mg/g), F represents the fraction of solute adsorbed at any time t, B_t_ is a mathematical function of F, was calculated for each value of F employing Reichenburg equation:

7 $$ {B}_t=-0.4977- \ln \left(1-F\right) $$

The linearity test of B_t_ vs. time plots was employed to distinguish between the film diffusion- and particle diffusion-controlled adsorption. If the plot of B_t_ vs. time (having slope B) is a straight line passing through the origin, then the adsorption rate is governed by particle diffusion mechanism otherwise it is governed by film diffusion [25]. The B_t_ vs. time plot was linear for benzene sorption up to 80 min from the aqueous solution onto RHC. The straight line does not pass through the origin, which confirms the observed behavior of the Weber–Morris model. Thus, particle diffusion is the most probable operating mechanism and does not control the kinetics of benzene sorption, i.e. external mass transport mainly governs the rate-limiting.

### Mechanism of benzene removal by RHC

It is of great importance to reveal the dominant adsorption mechanism of benzene on RHC even though it is not straightforward. In the adsorption mechanism of aromatic compounds in the liquid phase on activated, carbons there are two main types of interactions: electrostatic and dispersive [26].

Since benzene is in the molecular form in the solution, the adsorption mechanism may occur by the dispersive attraction between the π orbital of graphene sheet on the carbon basal planes and the electronic density in the benzene aromatic rings (π–π interactions) [1]. Furthermore, the electrostatic interaction between benzene molecules and the RHC surface may also explain the observation of high benzene adsorption on RHC. Jankowska and co-workers suggested that benzene can also be adsorbed on the weakly acidic or nonacidic oxygen groups by the interaction of benzene ring π electrons with the positive charge of those groups, which results in more electrostatic attraction and thus leads to a higher benzene adsorption process.

### Comparative study

The comparisons in benzene equilibrium adsorption capacity of various adsorbents including single-walled CNT (SWCNT), multiwalled CNT (MWCNT), PAC, GAC, montmorillonite, zeolite, and activated carbon fiber (ACF) conducted in this study or reported in the literature are given in Table 2. The present RHC shows better performance of benzene adsorption than many types of adsorbents. This suggests that the RHC are efficient benzene adsorbents. Since the production and the use of RHC have very low costs, thus, the RHC possesses a good potential for benzene removal in wastewater treatment.

**Table 2:** **Comparisons in benzene adsorption of various adsorbents**

**Adsorbent**	**q**_**e**_ **mg/g**	**Ref**
RHC	365	This study
AC	274.7	[27]
CNT	247.87	[28]
GAC	217.32	[28]
GAC	183.3	[1]
synthetic zeolites	150.42	[29]
GAC	114.4	[1]
ACF	66	[30]
SWCNT	60.1	[31]
PAC	40	[32]
MWCNT	36.2	[31]
Montmorillonite	28	[32]
Zeolite	27	[32]
synthetic zeolites	14.95	[33]

### Experimental section

#### Chemical and reagents

All chemicals used in the study are of high purity and are obtained from Sigma and Merck. All glassware was washed with HNO_3_ and distilled water and dried in an oven. Analytical grade benzene with >99% purity was purchased from Merck. A stock solution in methanol (Sigma-Aldrich, puriss p.a. >99.8%-GC) was prepared in 10 mL volumetric flasks containing 2000 ppm from each of the above-mentioned contaminants, using microliter syringes. The aqueous standard was prepared by spiking a measured quantity of methanol standard into a 100 ml volumetric flask filled with reagent water. These solutions were used for the kinetics experiments.

### Activated carbon preparation

Rice husk was obtained from local rice mills and was washed several times with bi-distilled water followed by filtration. Rice husk carbon (RHC) was prepared according to procedures in previous work with some modification [34]. The rice husk was mixed with phosphoric acid (50 wt%) in a mass ratio (rice husk to phosphoric acid) of 1:4, then the mixture was slightly agitated to ensure the penetration of the acid throughout, then the mixture was heated to 80°C for 1 h and left overnight at room temperature to help the appropriate wetting and impregnation of the precursor. The impregnated mass was heated gradually up to 700°C within 1 h in a steel pipe (furnace tube) and soaked at this temperature for two hours. After cooling, the carbonized mass was washed several times with bidistilled water (95°C) until pH 6.5 and dried at 110°C. The resulting solid product was named as RHC.

### Kinetic study

Kinetic experiments were carried out at room temperature using 110 mL glass bottles with an addition of 50 mg of RHC and 100 mL of benzene solution of initial concentrations (C_0_) of 200 mg/L, which were chosen to be representative of maximum benzene concentration in industrial wastewater. The mixture was filtered and C_e_ was measured after different intervals of time (0-300 min) until the residual concentration remained constant. Benzene concentration before and after adsorption were determined by Purge & Trap (model: HP-7695) gas chromatography (model: HP-6890) with flame ionization detector (PT-GC-FID) according to the US-EPA (524.2) method [35]. The sorption capacity of adsorbent was calculated by:

8 $$ \mathrm{qe}=\mathrm{V}\left({\mathrm{C}}_{\mathrm{o}}\hbox{-} {\mathrm{C}}_{\mathrm{e}}\right)/\mathrm{m} $$

where C_o_ and C_e_ are the initial and equilibrium concentration (mg/l) respectively, M is the mass of dry carbon sample used (g) and V volume of solution (ml).

## Conclusion

The adsorption of benzene using activated carbon prepared from rice husks (RHC) was investigated. The kinetic study of benzene on RHC was performed based on pseudo-first-order, pseudo-second-order, Elovich and intraparticle diffusion equations. The adsorption kinetics data followed the pseudo-second-order kinetic model, which suggests chemisorption nature of the process. The intraparticle diffusion model confirmed that intraparticle is not a fully operative mechanism, whereas the Boyed model suggested that film diffusion is the rate limiting step. A comparative study on benzene adsorption revealed that the RHC show superior adsorption performance as compared to many types of other adsorbents reported in the literature. Overall, RHC may be used as a low-cost, natural and abundant source for the removal of benzene from water and wastewater. Further studies on quantitative characterization of this adsorbent and involved mechanisms and the feasibility of using this adsorbent for other organic and inorganics for possible industrial application are needed.
